# Social relationships, mental health and wellbeing in physical disability: a systematic review

**DOI:** 10.1186/s12889-017-4308-6

**Published:** 2017-05-08

**Authors:** Hannah Tough, Johannes Siegrist, Christine Fekete

**Affiliations:** 1 Swiss Paraplegic Research, Guido A. Zäch Strasse 4, 6207 Nottwil, Lucerne, Switzerland; 2 Department of Health Sciences and Health Policy, University of Lucerne, Frohburgstrasse 3, P.O. Box 4466, 6002 Lucerne, Switzerland; 3 Senior Professorship ‘Work Stress Research’, Faculty of Medicine, University of Düsseldorf, Life-Science-Center, Merowingerplatz 1a, 40225 Düsseldorf, Germany

**Keywords:** Social relationships, Social support, Social networks, Mental health, Depression, Wellbeing, Physical disability

## Abstract

**Background:**

Research has consistently found that favourable exchange with one’s proximal social environment has positive effects on both mental health and wellbeing. Adults with physical disabilities may have fewer opportunities of favourable exchange, and therefore the effects on mental health and wellbeing may be less advantageous. The aim of this study is to systematically review quantitative studies exploring associations of social relationships with mental health and wellbeing in persons with physical disabilities.

**Methods:**

The databases PubMed, PsycINFO and Scopus were searched for relevant studies published between 1995 and 2016. Data was extracted on study and participants’ characteristics, independent and dependent variables, used measures and effects sizes of associations between social relationships and mental health or wellbeing. A narrative review was performed to synthesize findings along the constructs social support, social networks, negative social interactions, family functioning and relationship quality.

**Results:**

Of the 63 included studies, 47 were cross-sectional and 16 longitudinal. Most studies included a measure of social support (*n* = 58), while other concepts were less often studied (social networks *n* = 6; negative social interaction *n* = 3; family functioning *n* = 2; relationship quality *n* = 1). Over half of studies included depression as outcome (*n* = 33), followed by wellbeing (*n* = 14), composite mental health measures (*n* = 10), anxiety (*n* = 8), psychological distress (*n* = 7), posttraumatic stress disorder (*n* = 3), and hopelessness (*n* = 1). Although trends for associations of social support with mental health and wellbeing were consistent, around a quarter of studies failed to report significant associations. Social networks were related to depression, but not to other mental health or wellbeing measures. Family functioning, negative social interactions and relationship quality showed consistent associations with mental health and wellbeing, however, only few studies were available.

**Conclusions:**

This review indicates that social relationships play an important role in mental health and wellbeing in persons with disabilities, although findings are less consistent than in general populations and strength of associations vary between constructs. Integrating persons with disabilities into social networks seems not sufficient and rehabilitation professionals together with affected persons and their peers should ensure that high quality relationships and tailored support are available.

## Background

Disability is a growing public health problem, not least in ageing populations worldwide [1]. People with functional limitations or bodily impairments are generally disadvantaged in their opportunities to participate in social life [2]. These restrictions not only contradict basic human rights [3], but may also affect people’s health and wellbeing. There is consistent evidence that continued favourable exchange with one’s proximate social environment (e.g. family, friends and work life) exerts beneficial effects on health and wellbeing [4]. Conversely, social isolation or lack of close social ties is associated with poor health and increased mortality risk [5]. These associations hold true for the general population but are particularly relevant for persons with physical disabilities, due to their restricted social participation [6]. Reduced mental health in terms of psychiatric disorders is one of the major burdens of disease worldwide [7] and in particular in populations with disabilities [8].

There is convincing evidence that poor social relationships negatively impact mental health [9, 10]. So far, systematic reviews have summarized the links between social relationships and mental health in able-bodied populations [10], yet, no systematic review has been performed to document the current state of research in persons with physical disabilities. Traditionally, mental health is understood as a multidimensional construct of disease orientated symptoms [11, 12]. Given the pervasive effects of disability on major areas of everyday life, it is important to consider the subjective appraisal of one’s wellbeing. More precisely, wellbeing defined as the subjective appraisal of one’s functioning, mood and satisfaction with life complements the concept of mental health to represent this important dimension [13]. This review incorporates this distinction by analyzing the associations of social relationships separately for mental health and wellbeing.

Given the variety of concepts and measures of assessing social relationships, we first define the leading concepts as a prerequisite to structure the bulk of information provided by the extensive body of empirical data. The term *social relationships* encompasses a wide variety of aspects relating to the proximal and distal social environment. Distal environment includes the broader social structure of opportunities for social integration (e.g. cultural, labour market, neighbourhood) and its quality (e.g. social capital) [9, 14]. Aspects of the distal social environment are excluded from this review as direct effects on health and wellbeing are usually weak or absent after analyzing their mediation through proximal factors [15], and as evidence for populations with disabilities is widely lacking. Our work therefore focuses on two leading sociological concepts that analyse proximal factors of social relationships, namely social networks and social support [14]. *Social networks* describe the size, density, frequency and duration of social contacts [16], whereas *social support* emphasizes the functional significance in terms of providing instrumental, emotional or informational resources [17]. Important further aspects concern the quality of and satisfaction with support received and the distinction between perceived and received support. Further aspects look at the dynamics of specific relationships, for example the *relationship quality* [18] of dyadic couples or *family functioning* [19, 20]. Not all social interactions result in positive relationships and negative social interactions will also form a part of this review [21]. Finally the notion of *loneliness* is relevant in this context because the subjective feeling that it represents may have adverse effects on mental health and wellbeing, even in the presence of social contacts [22, 23]. We explore these aspects of social relationships from the perspective of persons with physical disabilities in order to assess how their perception of their interaction with the social environment is associated with mental health and wellbeing. Aspects of informal caregiving are not explicitly included in this review but may arise due to the inclusion of family functioning and received social support.

The objective of this review is thus to summarise a complex and heterogeneous body of empirical research on the association of different social relationship constructs with mental health and wellbeing in physical disability by and to highlight conceptual and methodological deficiencies in the field of research.

## Methods

### Search strategy

The literature search included original articles published in English between January 1, 1995 and May 31, 2016. This time frame was selected due to feasibility issues and in order to assess the contemporary social environment. Moreover, a selective screening of the literature before 1995 showed that the main findings of these studies fully support the conclusion of our review and therefore would not provide a significant extension of knowledge. The databases PubMed, PsycINFO and SCOPUS were searched. SCOPUS is worldwide the largest abstract and citation database of peer-reviewed literature and PubMed and PsycINFO were used due to their relevance to the review’s objective and scope. To capture a comprehensive sample of relevant articles, we used multiple search terms for ‘social relationships’ and ‘physical disability’ including but not limited to the terms *interpersonal relations, social environment, social isolation, social networks, relationship quality* and *disabled persons, activities of daily living, functional limitations, chronically ill*. We also included search terms for the following common health conditions *spinal cord injury, stroke, multiple sclerosis, rheumatoid arthritis* and *Parkinson’s disease*, as many studies on persons with disabilities identify specific health conditions in their keywords rather than general terms relating to disability (see Appendix 1 for full search strategy). The disability terms were intended to identify papers where the study population had functional limitations in activities of daily living due to physical impairments or mobility restrictions, and not study populations which were restricted due to intellectual, developmental or mental impairments. We only included study populations with a diagnosed health condition leading to a disability, for example, studies on general ageing populations were excluded. The social relationship term did not include aspects associated with the distal social environment, such as culture, social capital and social cohesion as explained in the Background [14].

As a quality assessment for reporting, the PRISMA statement was adopted [24].

### Inclusion and exclusion criteria

Original studies were eligible if they provided quantitative data regarding adults’ (≥ 18 years old) mental health or wellbeing outcomes as a function of social relationships, including both functional and structural aspects. In line with the literature, the term mental health was used to address health conditions (ICD-10 defined conditions, e.g., major depression) as well as mental functioning (e.g., SF-36 mental health subscale). Wellbeing was defined as a multidimensional concept which contains subjective appraisals of different aspects of life, including but not restricted to health. In line with the traditional understanding of subjective wellbeing described by Diener et al. [13, 25], we included studies that used the concept of wellbeing related to how individuals experience their quality of life including emotional reactions and cognitive evaluations of the satisfaction with general and specific life areas. Some studies subsumed established measures on general health as wellbeing or quality of life. We have only included these studies if they reported on a component of mental health. Mental health and wellbeing were not included in the search strategy in order to avoid potentially relevant studies being overlooked.

**Fig. 1:**
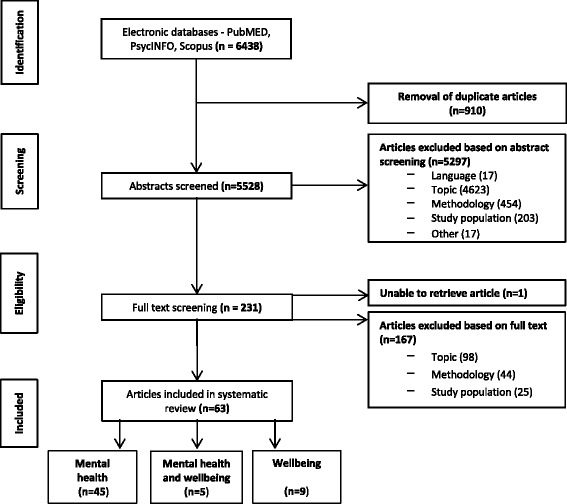
Flowchart of studies excluded and selected for systematic review

After removing duplicates (*n* = 910) and studies not in English language (*n* = 17), 5528 abstracts were screened based on predefined inclusion and exclusion criterion concerning topic, methodology, and study population. We excluded studies on a thematic basis if the topic was irrelevant to social relationships and health, if social relationships were not treated as an independent variable or if social relationships were not associated to a mental health or wellbeing outcome (*n* = 4623). We excluded studies on a methodological basis if the sample size was below 50, if social relationships were not assessed by a validated measurement instrument, if qualitative methodology was applied or if the article was an editorial or a review (*n* = 454). These criteria were applied to ensure that included studies were comparable and that studies with limited statistical power due to a low sample size were excluded. The inclusion of only those studies using validated instruments i.e. those psychometrically tested, aimed to ensure that social relationship concepts were adequately measured. Furthermore, studies focussing on persons other than those with physical disabilities (e.g., caregivers, health care providers) or persons with intellectual, developmental or mental disabilities were excluded (*n* = 203). In total, *n* = 231 full-text articles were screened for inclusion and *n* = 63 articles were included in our review. After full-text screening, *n* = 98 studies were excluded based on the topic, *n* = 44 based on methodology and *n* = 25 based on the study population (Fig. 1).

HT screened all abstracts ascertaining the relevance of the study and applying the inclusion and exclusion criteria. Double screening was performed on 5% of abstracts due to feasibility and a high level of reviewer agreement (94%). In case of uncertainty, the full-text was obtained and included in the full-text screening. HT screened all full-texts and 20% of full-texts were double screened by CF, with a reviewer agreement of 100%. If there was any indecision about the inclusion of an article this was discussed and if necessary, a third reviewer was consulted (JS).

### Data extraction

To standardise data collection, a Microsoft Access 2010 database was created to extract the following information: First author, year of publication, country, sample size, study design, participant characteristics (age, gender, disability), independent and dependant variables, measurement instruments, effect sizes from bivariate and multivariable analyses, measure of variance and confounding variables controlled for. When several models presenting different confounders were reported, the fully-adjusted models were selected for data extraction. Multiple effect sizes were extracted from those studies that measured several associations between different aspects of social relationships and mental health or wellbeing. Effect sizes from every time point in longitudinal studies were extracted. When studies reported results separately for sub-groups of participants, the specific findings for each sub-group were recorded along with overall results. For quality assurance, data extraction was conducted by two independent reviewers for 10% of the included studies. Reviewer agreement here was 100%.

### Quality assessment

All included studies were assessed independently for quality of reporting by HT and CF using the Strengthening the Reporting of Observational studies in Epidemiology (STROBE) guidelines [26]. STROBE is a quality assessment tool for observational studies which consists of 22 criteria to evaluate the reporting of the background, study design, data collection and data analysis of the study. This resulted in a score for each study ranging from lowest quality (0) to highest quality (22). For ease of interpretation the STROBE score was converted into a low (8–11), medium (12–15) and high (16–22) quality rating.

### Analysis

Study and participants characteristics of included studies are described (Table 1). To synthesise evidence, the details of each study including country, sample size, measures of social relationships and mental health or wellbeing, quality rating, participant characteristics and key findings were entered in Table 2 for cross-sectional and Table 3 for longitudinal studies. Key findings of cross-sectional studies are reported by declaring the variables under study, the direction of the association (+ for positive association; – for negative association; 0 for no association) and whether the association was statistically significant or not (+ and – indicate statistically significant results with *p* ≤ 0.05; + or – in brackets indicate non-significant results). Given the complexity of reporting longitudinal results, key findings of prospective studies were described in text form.

**Table 1:** Study and participant characteristics of included studies

	Category specification	N (%) or mean (range)
Total included		63 (100%)
Study characteristics		
*Region*	Europe	32 (50.8)
	North America	23 (36.5)
	Asia	7 (11.1)
	Australasia	1 (1.6)
*Design*	Cross-sectional	47 (74.6)
	Longitudinal	16 (25.4)
*Social relationship concepts*	Social support	58 ^a^
	Social network	6
	Negative social interactions	3
	Family functioning	2
	Relationship quality	1
*Mental health*	Depression	33 ^a^
	Mental health composite score	10
	Anxiety	8
	Distress	7
	Posttraumatic stress disorder	3
	Hopelessness	1
*Wellbeing*	Life satisfaction	6 ^a^
	Quality of life	6
	Negative affect	3
	Positive affect	2
	Wellbeing	2
*Quality rating* ^*b*^	Low	15 (23.8)
	Medium	34 (54.0)
	High	14 (22.2)
Participant characteristics		
*Sample size*	Mean, range	232.5 (50–1455)
*Age*	Mean, range	52.1 (21.0–75.6)
*Health condition*	Rheumatoid arthritis	22 (34.9)
	Spinal cord injury	14 (22.2)
	Multiple sclerosis	12 (19.0)
	Stroke	11 (17.4)
	Physically disabled, unspecified	2 (3.2)
	Spina bifida	1 (1.6)
	Parkinson’s disease	1 (1.6)

^a^Percent of studies not given as some studies assess more than one social relationship concept and/or include several mental health or wellbeing outcomes

^b^Quality of reporting assessed by the STROBE guidelines

**Table 2:** Cross-sectional studies on social relationships and mental health and/or wellbeing in disability

First author, year, reference	Country	Social relationship measures	Mental health/wellbeing measures	Quality rating	Participant characteristics				Key findings ^a^
					*N*	Mean age	% males	Disability	
Social support									
Abraido-Lanza 2004 [33]	USA	Social support *SSQS*	Psychological wellbeing *PANAS*, Depression *CES-D*	Medium	98	50.6	0.0	Rheumatic disease	Emotional support / Wellbeing + Instrumental support / Depression -
Agar 2006 [99]	UK	Social support *SSQ-6*	PTSD *IES, PDS*	Medium	50	38.9	86.0	Spinal cord injury	^b^
Bambara 2011 [54]	USA	Social support, positive interaction *MOS SSS*	Depression *PHQ-9*	High	451	55.1	86.4	Multiple sclerosis	Perceived social support / Depression -
Bamer 2008 [39]	USA	Social support *MOS SSS*	Depression *CES-D*	High	530	54.2	25.0	Multiple sclerosis	Lack of social support / Depression +
Beedie 2002 [55]	UK	Social support *SSQ-6*	Depression *BDI*, Suicidal ideation, hopelessness*, BHI*	Medium	100	30.0	79.2–85.7	Spinal cord injury	Satisfaction social support / Depression - Satisfaction social support / Hopelessness -
Cheng 2008 [29]	China	Social support *SSRS*	Depression *HAMD*	Medium	121	65.2	69.4	Parkinson’s disease	Received social support / Depression -
Coty 2010 [37]	USA	Social support *STMSSC Problematic support*	Life satisfaction *SWLS,* Depression *CES-D*, Negative affect *PANAS*	Low	73	57.0	0.0	Rheumatoid arthritis	Negative social support / Depression + Negative social support / Negative affect + Negative social support / Life satisfaction (−) Unavailability social support / Depression + Unavailability social support / Negative affect + Unavailability social support / Life satisfaction (−)
Danner 2000 [40]	USA	Social support *PSSS*	PTSD *SCID, CAPS, IES*	Medium	124	48.8	100.0	Spinal cord injury	Family social support / PTSD (+) Family social support / IES (−) Friends social support / PTSD - Friends social support / IES (−)
Dirik 2009 [43]	Turkey	Social support *MSPSS*	Depression, anxiety *HADS*	Low	117	48.5	15.4	Rheumatoid arthritis	Perceived social support / Anxiety (−) Perceived social support / Depression (−)
Dodd 2015 [44]	USA	Social support *SPS*	Depression *PHQ-9*	Medium	106	43.8	64.2	Spinal cord injury	Social support / Depression (+)
Dwyer 1997 [94]	USA	Social support *ISEL*	Affective distress *AIMS*	Low	185	43.0	0.0	Rheumatoid arthritis	Social support / Affective distress +
Fyrand 1997 [61]	Norway	Social support, social companionship *SSQT*	Anxiety, depression *GHQ*	Low	138	55.0	0.0	Rheumatoid arthritis	Social support / Depression (−) Social companionship / Depression - Social support / Anxiety 0 Social companionship / Anxiety 0
Gay 2010 [56]	France	Social support *SSQ-6*	Depression *ADS,* Anxiety *STAI*	Medium	115	47.2	31.3	Multiple sclerosis	Satisfaction with social support / Depression -
Geuskens 2006 [66]	Netherlands	Social support ISS	Mental health *SF-36*	High	359	49.9	27.4	Inflammatory joint complaints	Social support / Mental health +
Gottlieb 2001 [72]	Israel	Social support *Tel-Aviv SSI*	Life satisfaction *LSI*	Low	100	73.0	59.0	Stroke	Social support / Life satisfaction +
Hampton 2008 [73]	China	Social support *PSSS*	Subjective wellbeing *IPW*	High	119	25.0	61.0	Spinal cord injury	Perceived social support / Affective wellbeing +
Hatcher 2009 [48]	UK	Social support *PSSS*	PTSD *IES, PANAS, PTCI*	Medium	102	45.7	81.4	Spinal cord injury	^b^
Hilari 2006 [49]	UK	Social support *MOS SSS*	HRQoL *SAQOL-39*	Medium	83	61.6	92.7	Stroke	^b^
Huang [46]	Taiwan	Social support *Modified social support inventory*	Depression *BDI*	High	135	43.3	83.0	Spinal cord injury	Social support / Depression 0
Jaracz 2010 [50]	Poland	Social support *SPS*	Mental health *MSQOL-54*	Medium	210	37.4	28.6	Multiple sclerosis	^b^
Jensen 2014 [42]	USA	Social support *MSPSS*	Depression *PHQ-9*	Medium	1416	52.6	41.0	Multiple sclerosis, spinal cord injury, muscular dystrophy	Family social support / Depression (−) Friends social support / Depression - Significant other social support / Depression (−)
Kim 1999 [51]	Canada	Social support *SSIPAD*	Quality of life *QLI-Stroke version*	High	50	75.0	58.0	Stroke	^b^
King 1996 [100]	USA	Social support *SSE*	Quality of life *QLI*	Medium	86	63.3	65.0	Stroke	Perceived social support / Quality of life +
Kivisild 2014 [52]	Estonia	Social support *SSQ*	Mental health *RAND-36*	Medium	80	38.9	82.5	Spinal cord injury	^b^
Kool 2013 [67]	Netherlands and Belgium	Social support *MOS SSS,*	Mental health *SF-36*	Medium	1455	46.2	14.0	Rheumatic disease	Perceived social support / Mental health +
Kraaimaat 1995 [65]	Netherlands	Social support *IRGL*	Depression, anxiety *IRGL*	Medium	229	58.6	42.4	Rheumatoid arthritis	MEN Social support / Anxiety (−) Social support / Depression (−) WOMEN Social support / Anxiety - Social support / Depression (−)
Krokavcova 2008 [41]	Slovakia	Social support *PSSS*	Mental health *SF-36*	Medium	207	38.4	33.8	Multiple sclerosis	Family social support / Mental health + Friends social support / Mental health + Significant other social support / Mental health (+)
Lewin 2013 [57]	Germany	Social support *F-SozU*	Depression *GDS*	Low	96	67.1	52.0	Stroke	Perceived social support / Depression -
Müller 2015 [63]	Switzerland	Social support *SSQ 6*	Depression *HADS* Quality of life *WHOQoL BREF*	High	503	54.6	71.8	Spinal cord injury	Social support / Depression (−) Social support / Quality of life +
Osborne 2007 [68]	USA	Social support *MSPSS*	Mental health *SF-36*	Medium	125	50.8	24.8	Multiple sclerosis	Social support / Mental health +
Phillips 2009 [58]	USA	Social support *PRQ2000*	Depression *CES-D*	Low	118	53.26 FM 45.53 MS	0.0	Multiple sclerosis, fibromyalgia	Social support / Depression -
Pitsilka 2015 [75]	Greece	Social Support *QSSS*	Quality of Life *RaQoL*	High	127	60.7	16.5	Rheumatoid arthritis	Social support / Quality of life +
Raichle 2007 [69]	USA	Social support *MSPSS WHYMPI*	Mental health *SF-36*	Medium	157	48.5	72.4	Spinal cord injury	Perceived social support / Mental health +
Riemsma 2000 [38]	Netherlands	Social support *SSL Problematic support*	Depression *AIMS 2*	Low	229	62.7	39.0	Rheumatoid arthritis	Social support / Depression - Problematic social support / Depression +
Rintala 2005 [64]	USA	Social support *SF-ISEL*	Depression *CES-D*, Anxiety *STAI*, Life satisfaction *SWLS, RAND-36*	Low	165	55.0	100.0	Spinal cord injury	Social support / Anxiety (−) Social support / Depression (−) Social support / Life satisfaction +
Ritvo 1996 [70]	Canada	Social support *SF-ISEL*	Mental health *MHI*	Medium	130	41.86	13.0	Multiple sclerosis	Social support / Mental health +
Schwartz 2005 [71]	Israel	Social support *MOS SSS*	Mental health *QoL MSQLI*	Low	82	45.1	79.0	Multiple sclerosis	Perceived social support / Mental health +
Shao 2014 [47]	China	Social support *SSQT*	Subjective wellbeing *SWS*	Medium	214	70.3	59.8	Stroke	Social support / Subjective wellbeing 0
Stroud 2006 [45]	USA	Social support *SSQ-6*	Depression *CES-D*	Medium	70	46.0	64.0	Spinal cord injury	Number social support / Depression (−) Satisfaction social support / Depression (−)
Stuifbergen 2009 [101]	USA	Social support *PRQ*	Quality of life *QLI*	Medium	442	55.9	16.0	Multiple sclerosis	Perceived social support / Quality of life +
Suh 2012 [59]	USA	Social support *SPS*	Depression *HADS*	Medium	218	43.5	10.0	Relapse remitting multiple sclerosis	Social support / Depression -
Suurmeijer 2005 [34]	Netherlands	Social support, social companionship *SSQT*	Anxiety, depression *GHQ-28*	Medium	280	53.4	36.0	Rheumatoid arthritis	Informational social support / Anxiety – Social companionship / Depression -
Treharne 2005 [53]	UK	Social support *MOS SSS*	Depression, anxiety *HADS,* Life satisfaction *QoLS*	Medium	154	56.3	27.0	Rheumatoid arthritis	^b^
Wu 2007 [102]	China	Social support *PSSS*	Depression *CES-D*	Low	204	44.23	55.4	Physically disabled	Perceived social support / Depression -
Zhang 2011 [30]	China	Social support *SSRS*	Depression *GDS*	Medium	81	>60	56.0	Stroke	^b^
Social network									
Berkanovic 1996 [86]	USA	Social network *LSNS*	Depression *HAQ*	Low	118	51.7	25.0	Rheumatoid arthritis	Social network / Depression -
Kraaimaat 1995 [65]	Netherlands	Social network *IRGL*	Depression, anxiety *IRGL*	Medium	229	58.6	42.4	Rheumatoid arthritis	MEN Social network / Anxiety (−) Social network / Depression - WOMEN Social network / Anxiety 0 Social network / Depression 0
Nicassio 2011 [87]	USA	Social network *SNI*	Mental health *SF-36*	High	106	56.2	17.0	Rheumatoid arthritis	Social network / Mental health 0
Pitsilka 2015 [75]	Greece	Social network *SNI*	QoL *RaQoL*	High	127	60.7	16.5	Rheumatoid arthritis	Social network / Quality of life 0
Family functioning									
Bellin 2010 [88]	USA	Satisfaction with family functioning *The Family APGAR*	Depression, anxiety *HSCL-25*	High	61	21.0	39.3	Spina bifida	Satisfaction family functioning / Depression – Satisfaction family functioning / Hopelessness (−)
Coty 2010 [37]	USA	Family functioning *FRI*	Life satisfaction *SWLS*, Depression *CES-D*, Negative affect *PANAS*	Low	73	57.0	0.0	Rheumatoid arthritis	Family functioning / Depression – Family functioning / Negative affect – Family functioning / Life satisfaction +
Negative social interactions									
Kool 2013 [67]	Netherlands and Belgium	Negative responses *III*	Depression *BDI,* Mental health *SF-36*	Medium	1455	46.2	14.0	Rheumatic disease	Discounting / Mental health – Lack of understanding / Mental health –
Kraaimaat 1995 [65]	Netherlands	Reaction of spouse *IRGL*	Depression, anxiety *IRGL*	Medium	229	58.6	42.4	Rheumatoid arthritis	MEN Criticism / Anxiety + Criticism / Depression (+) Distraction / Anxiety (+) Distraction / Depression (+) WOMEN Criticism / Anxiety + Criticism / Depression + Distraction / Anxiety (+) Distraction / Depression (+)
Stroud 2006 [45]	USA	Partner response to pain *MPI*	Depression *CES-D*	Medium	70	46.0	64.0	Spinal cord injury	Negative partner response to pain / Depression +
Relationship quality									
McPheters 2010 [89]	USA	Partner relationship quality *DAS*	Depression *CES-D, PHQ-9*	Medium	54	53.2	20.0	Multiple sclerosis	Relationship quality / Depression -

^*a*^ *Key findings are from multivariate results. + indicates significant positive association (p ≤ 0.05); − significant negative association (p ≤ 0.05), 0 no association; (+) positive but non-significant trend; (−) negative but non-significant trend.* ^*b*^ *No multivariate results available*

*Abbreviations for social relationship measures* *:* *DAS* Dyadic Adjustment Scale*,* *FRI* Family Relationship Index, *F-SozU* Fragebogen zur sozialen Unterstützung*,* *III* The Illness Invalidation Inventory, *IRGL* Impact of Rheumatic Diseases on General Health and Lifestyle, *ISEL* Interpersonal Support Evaluation List, *ISS* Inventory of Social Support, *LSNS* Lubben Social Network Scale, *MOS SSS* Medical Outcomes Study Social Support Scale, *MPI* Multidimensional Pain Inventory, *MSPSS* Multidimensional Scale of Perceived Social Support, *PRQ* Personal Resource Questionnaire, *PSSS* Perceived Social Support Scale, *PDS* Posttraumatic Diagnostic Scale, *SNI* Social Network Index, *SPS* Social Provisions Scale, *SSE* Social Support Effectiveness, *SSIPAD* Social Support Inventory for People with Acquired Disabilities, *SSL* Social Support List, *SSQ-6* Social Support Questionnaire, *SSQS* Social Support Questionnaire for Satisfaction, *SSQT* Social Support Questionnaire for Transactions; *SSRS* Social Support Resource Scale, *STMSSC* Stong Ties Measure Social Support Scale, *Tel-Aviv SSI* Tel-Aviv Social Support Instrument, *QSSS* The Quality of Social Support Scale

*Abbreviations for mental health and wellbeing measures* *:* *ADS* Anxiety Depression Self-rating Scale, *AIMS* Arthritis Impact Measurement Scale, *BDI* Beck Depression Inventory, *BHI* Beck Hopelessness Inventory, *CAPS* Clinician Administered PTSD Scale, *CES-D* Centre for Epidemiological Studies Depression, *GDS* Geriatric Depression Scale, *GHQ* General Health Questionnaire, *HADS* Hospital Anxiety and Depression Scale, *HAMD* Hamilton Depression Scale, *HAQ* Health Assessment Questionnaire, *HSCL-25* The Hopkins Symptom Checklist, *IA* Index of Affect, *IES* Impact of Event Scale, *IPW* Index of Psychological Wellbeing, *IRGL* Impact of Rheumatic Diseases on General Health and Lifestyle, *LSI* Life Satisfaction Index, *MHI* Mental Health Inventory, *MSQLI* Multiple Sclerosis Quality of Life Index, *MSQOL-54* Multiple Sclerosis Quality of Life, *PANAS* Positive and Negative Affect Scale, *PHQ-9* Patient Health Questionnaire-9, *PTCI* Post Traumatic Cognitions Inventory, *QLI* Quality of Life Index, *QOLS* Quality of Life Scale, *RAND-36* Medical Outcomes Survey, *RaQOL* Rheumatoid Arthritis Quality of Life Questionnaire, *SAQOL-39* Stroke and Aphasia Quality of Life Scale, *SCID* Structured Clinical Interview for DSM Disorders, *SF-36* Short Form Health Survey, *STAI* State Trait Anxiety Inventory, *SWLS* Satisfaction with Life Scale, *SWS* The Subjective Wellbeing Scale, *WHOQOL-BREF* World Health Organisation Quality of Life-BREF

**Table 3:** Longitudinal studies on social relationships and mental health and/or wellbeing in disability

First author, year, reference	Country	Follow up time, number of waves	Social relationship measures	Mental health / wellbeing measures	Quality rating	Participant characteristics					Key findings ^a^
						*N*	Mean age	% males	Disability	Disease duration	
Social support											
Benka 2012 [35]	Slovakia (EURIDISS)	4 years, 4	Social support *SSQS*	Psychological distress *GHQ-28*	Medium	116	47.6	15.5	Rheumatoid arthritis	0–4 years after diagnosis	Emotional and instrumental social support increased over time. Emotional support T1-T3 significantly negatively associated with psychological distress at T4. Instrumental support T1-T3 not associated with distress at T4.
Costa 2013 [81]	Portugal	2 years, 2	Social support *AIMS 2*	Depression *DASS*	Medium	55	55.2	20.0	Rheumatoid arthritis	First 2 years of disease progression	Low social support at T1 positively associated with depression at T2.
Curtis 2004 [84]	Ireland	1 year, 2	Social Support *MOS SSS*	Anxiety, depression *AIMS* Positive and negative affect *PANAS*	Low	52	60.0	0.0	Rheumatoid arthritis	13 years	Cross-sectional analyses at T1 and T2 showed no significant association of perceived social support with depression, anxiety, positive affect or negative affect when controlling for disease status and perceived stress.
Demange 2004 [78]	France, the Netherlands, and Norway (EURIDISS)	3 years, 3	Social support, social companionship *SSQT*	Psychological distress *GHQ*	High	542	52.5	31.0	Rheumatoid arthritis	0–4 years after diagnosis	Social support did not change over time. Cross-sectional relationships between social support and psychological distress were significant but no longitudinal within subject variation was associated with baseline social support or changes in social support over time. Social companionship decreased over time. Cross-sectional relationships between social companionship and psychological distress were significant but no longitudinal within-subject variation in distress was associated with baseline social companionship.
Doeglas 2004 [82]	Netherlands (EURIDISS)	3 years, 4	Social support, social companionship *SSQT*	Depression *GHQ*	High	264	53.0	35.0	Rheumatoid arthritis	0–4 years after diagnosis	Level of social support did not change significantly over time. Social support at T1 was significantly inversely associated with depression at T4. Social companionship at T1 did not show a significant association with depression at T4.
Evers 1997 [83]	Netherlands	1 year, 2	Social support *IRGL*	Anxiety, depression *IRGL*	Low	91	57.0	30.0	Rheumatoid arthritis	Shortly after diagnosis	Perceived social support at T0 was significantly inversely associated with anxiety and depression at T0 but not T1.
Evers 2002 [85]	Netherlands	5 years, 3	Social support *IRGL*	Anxiety, depression *IRGL*	Low	78	57.0	30.0	Rheumatoid arthritis	Shortly after diagnosis	Perceived social support at baseline was not significantly associated to depression at 3 or 5 years.
Hilari 2010 [79]	UK	6 months, 3	Social support *MOS SSS*	Psychological distress *GHQ*	High	87	69.3	56.0	Stroke	At stroke onset	Social support was significantly inversely associated with psychological distress at T1.
Sit 2007 [36]	Hong Kong	6 months, 2	Social support, social companionship *SSQT*	Depression *CES-D*	Medium	95	67.0	51.6	Stroke	At stroke onset	Information support and social companionship at T1 but not T0 were significantly negatively associated with depression at T1.
Strating 2006 [77]	Netherlands (EURISIDD)	8 years, 5	Social support, social companionship *SSQT*	Psychological distress *GHQ*	High	129	51.0	29.0	Rheumatoid arthritis	0–4 years after diagnosis	Social companionship did not change significantly over time whereas emotional support decreased significantly between T4 and T5. Both social support and social network (T1-T3) were insignificantly associated with psychological distress at T4 and T5, when distress at T1-T3 was entered into the model.
Townend 2007 [76]	Australia	3 months, 3	Social support *MSPSS*	Depression *HADS*	Medium	125	75.6	49.0	Stroke	At stroke onset	Social support increased over time. Social support at T1 and T3 was negatively associated with depression at T1 and T3.
Van Leeuwen 2010 [31]	Netherlands	1 year, 2	Social support *SSL-12*	Life satisfaction *NV*	Medium	190	40.6	74.7	Spinal cord injury	At the start of active rehabilitation	Everyday social support and support in problem situations decreased over time, whereas esteem support remained stable. Everyday social support was positively associated and support in problem situations was negatively associated to life satisfaction over time, in particular in persons with high levels of distress.
Van Leeuwen 2012 [32]	Netherlands	5 years, 3	Social support *SSL-12*	Life satisfaction *NV*	Medium	162	39.0	72.6	Spinal cord injury	At the start of active rehabilitation	Only everyday social support had a significant positive association with life satisfaction. Esteem social support and support in problem situations showed no significant association with life satisfaction.
Social network											
Evers 1997 [83]	Netherlands	1 year, 2	Social network *IRGL*	Anxiety, depression *IRGL*	Low	91	57.0	30.0	Rheumatoid arthritis	Shortly after diagnosis	Social network was not associated with anxiety or depression at T0 and only with depression at T1.
Evers 2002 [85]	Netherlands	5 years, 3	Social network *IRGL*	Anxiety, depression *IRGL*	Low	78	57.0	30.0	Rheumatoid arthritis	Shortly after diagnosis	Social networks at baseline was not significantly associated to depression at 3 or 5 years.
Relationship quality											
Robinson 1999 [90]	USA	2 years, 3	Social functioning *SFE*	Depression *HAMD*	Medium	50	60.0	66.0	Stroke	3–6 months after stroke onset	Relationship with significant other was significantly inversely associated with depression at T0. No measures of social functioning with significant other, family or children was associated with depression at T1 and T2.

EURIDISS: European Research on Incapacitating Diseases and Social Support

^*a*^ *Key findings are from multivariate results. + indicates significant positive association (p ≤ 0.05); − significant negative association (p ≤ 0.05), 0 no association; (+) positive but non-significant trend; (−) negative but non-significant trend*

*Abbreviations for social relationship measures*: *AIMS 2* Arthritis Impact Measurement Scale 2, *IRGL* Impact of Rheumatic Diseases on General Health and Lifestyle, *MOS SSS* Medical Outcomes Study Social Support Scale, *MSPSS* Multidimensional Scale of Perceived Social Support, *SFE* Social Functioning Examination, *SSL* Social Support List, *SSQS* Social Support Questionnaire for Satisfaction, *SSQT* Social Support Questionnaire for Transactions

*Abbreviations for mental health and wellbeing measures* *:* *AIMS* Arthritis Impact Measurement Scale, *CES-D* Centre for Epidemiological Studies Depression, *DASS* Depression, Anxiety and Stress Scale, *GHQ-28* GeneralHealth Questionnaire-28, *HADS* Hospital Anxiety and Depression Scale, *HAMD* Hamilton Depression Scale, *IRGL* Impact of Rheumatic Diseases on General Health and Lifestyle, *PANAS* Positive and Negative Affect Scale

Due to the heterogeneity of both independent and dependent variables, a meta-analysis was not feasible. Instead, the results from included studies were combined into a narrative synthesis to draw conclusions [27]. In comparison to meta-analysis, which uses statistical techniques to derive a pooled estimate of the effect size, narrative synthesis focuses primarily on the use of text to explain and summarise results from multiple studies. In this narrative synthesis, we grouped studies into thematically or conceptually related categories to study the amount of studies looking at certain themes and the number of associations between different social relationship constructs and mental health or wellbeing. We also considered the strength, direction, statistical significance and consistency of associations and additionally took into account potential change over time or differences in associations between subgroups.

## Results

Table 1 shows an overview of the social relationship, mental health and wellbeing constructs under study along with the participant characteristics of the included studies. The vast majority of the 63 included studies focussed on social support (*n* = 58), with relatively few other constructs being identified: Social networks were assessed in six studies, negative social interaction in three studies, family functioning in two studies, and relationship quality in one study. Concerning mental health and wellbeing constructs, depression was the most heavily studied construct being reported by 33 studies, a composite mental health score was reported by ten studies, followed by anxiety (*n* = 8), psychological distress (*n* = 7), posttraumatic stress disorder (*n* = 3), and hopelessness (*n* = 1). The health condition most prolifically studied was rheumatoid arthritis, particularly due to the inclusion of several studies from a large scale European-wide study focusing on social support in arthritis (EURIDISS) [28]. The mean STROBE sum score was 13.5, ranging from 8 to 19. The description of any efforts to address potential sources of bias (met by 9.5% of included studies) and the explanation of how the study size was arrived at (met by 7.9% of included studies) were the two STROBE criteria which were most frequently unmet.

Tables 2 and 3 provide a summary on characteristics and key findings of included cross-sectional and longitudinal findings, respectively. Results are presented along the social relationship constructs I) social support, II) social networks, III) negative social interactions, IV) family functioning and V) relationship quality. For all five constructs, we first present an overview on study characteristics (specification of constructs, measurements, study quality), followed by an in-depth discussion on cross-sectional and longitudinal findings.

I) *Social support*

Of the 58 studies focussing on social support, 45 were cross-sectional and 13 longitudinal. Social support constructs were operationalized heterogeneously, including type (emotional, instrumental, affective or tangible) or source (e.g., family, friends, significant other) of social support, overall measures of perceived or received social support, satisfaction with support, negative social support or unavailability of support. Four studies assessed received social support, defined as actual exchange of support [29–32]. Six studies report their results according to type of social support [31–36]. Two studies looked at negative or problematic social support [37, 38], two at the unavailability of social support [37, 39], and three studies distinguished between the source of social support (friend, family, significant other) [40–42]. The remaining 44 studies investigated perceived social support or satisfaction with support, although precise definitions of the terms under study were often missing. This heterogeneity of used constructs is reflected by the fact that we found a total of 21 different measures to assess social support. Overall, we did not observe any systematic association between study quality and strength of associations for studies including a social support measure.

*Cross-sectional findings.* 33 of the 45 cross-sectional studies found a significant association between social support and mental health and/or wellbeing, while three studies reported a non-significant trend [43–45], two studies showed no association [46, 47] and seven did not test the association in multivariable analyses [30, 48–53].

More specifically, of the 25 studies assessing *depression*, 14 found an inverse association between some element of social support and depression [29, 33, 34, 37–39, 42, 54–60], six studies reported a trend towards an inverse association [43, 45, 61–64], and one observed no association [46]. Two studies tested associations only in bivariable analyses and found no significant correlations [30, 53]. As mainly composite scores of social support were used, it was not possible to identify whether a certain aspect of social support was more protective than another. Of the four studies assessing *anxiety*, none reported a consistent association [53, 61, 64, 65]. A total of three studies looked at *posttraumatic stress disorder* (PTSD). While two of them only performed bivariable analysis [48, 99] one found a negative association between social support provided by friends and PTSD [40]. Of the nine studies assessing composite scores of *mental health*, seven found a positive association [41, 66–71]. Of the 14 studies assessing *wellbeing*, eight reported a positive association in multivariable analysis [33, 58, 63, 64, 72–75] and three studies did not report the results for multivariable analysis as bivariable associations were insignificant [49–51].

*Longitudinal findings.* Of the included longitudinal studies, 15 out of 16 involved a social support construct (Table 3). Results suggest that at very early stages of the disease process social support increased over time [35, 76] only to decrease at later stages [31, 77]. Four studies looked at social support’s association with *psychological distress* [35, 77–79]. Three of these studies found a change in psychological distress over time as a function of social support [77, 79, 80], whereas one study only found cross-sectional associations [78]. The one study only which distinguished between emotional and instrumental support observed associations of distress with emotional support but not with instrumental [35]. Seven studies analysed the association between social support and *depression*, two of these studies showed evidence of a longitudinal relationship between social support and depression [81, 82], i.e. earlier measures of social support effecting depression at later time points. Three studies only found cross-sectional associations [36, 76, 83] and two studies could not observe any association between social support and depression [84, 85]. The three studies addressing *anxiety* as an outcome showed no longitudinal associations with social support [83–85].

II) *Social networks*

Four cross-sectional studies included a measure of social networks as an independent variable (Table 2) [65, 75, 86, 87]. All measures of social networks addressed the size of network in terms of the frequency of interaction with different members or organisations of the network. Associations to *depression* were limited to men in one study [65] or to both men and women in a study of low quality [86]. There were no significant associations of social networks with a composite measure of *mental health* [87], *anxiety* [65], or *wellbeing* [75].

Of the included longitudinal studies, two out of 16 included social networks as an independent variable (Table 3) [83, 85]. Both studies assessed the same population at different time points and were of low quality. In both studies, social networks showed weaker associations with *depression* and *anxiety* than perceived social support. One study found a negative association between social network in terms of number of friends shortly after diagnosis and depression 1 year later [83].

III) *Negative social interactions*

Three cross-sectional studies looked at negative social interaction (Table 2) [45, 65, 67]. Aspects of negative social interaction included lack of understanding, criticism and negative spousal responses to pain. All of these studies were medium in quality, two of which solely focused on interactions within partnerships [45, 65], whereas one also investigated negative responses from different sources such as family members and colleagues [67]. All three studies provide evidence for associations with mental health: Negative associations between discounting, lack of understanding and mental health were found to be statistically significant [67], along with positive associations of criticism with anxiety [65], and of negative partner responses to pain with depression [45].

IV) *Family functioning*

Two studies looked at family functioning [37, 88], one of high quality [88] and one of low quality [37]. Inverse associations of family functioning with depression [37, 88] and negative affect [37], and positive associations with life satisfaction were found [37], but none with hopelessness [88]. Effect sizes of associations of family functioning with negative affect and depression were similar to those of social support, but somewhat larger for associations with life satisfaction [37].

V) *Relationship quality*

The one cross-sectional study which assessed relationship quality found an inverse relationship between relationship quality and depression [89]. This cross-sectional finding was supported in an additional longitudinal study, although there was no evidence of a longitudinal association [90]. Both studies were rated medium in quality.

## Discussion

In this review, we summarised a complex and heterogeneous body of empirical research on associations of social relationships with mental health and wellbeing in physical disability. When basing conclusions solely on results reported from multivariable analyses, we found consistent associations between social support and composite scores of mental health. The associations of social support with anxiety, depression and wellbeing were less pronounced as many studies reported insignificant associations. Remarkably, social networks seem to be related to depression, but not to any of the other studied measures on mental health or wellbeing. In contrast, family functioning, negative social interactions and relationship quality showed consistent associations with indicators of mental health and wellbeing, although the evidence was limited due to the small number of studies focusing on these concepts.

The aspect of social support is particularly dominant in disability research. Social support is considered a vital resource for hindering the negative consequences of a wide variety of stressors in disability (the ‘buffering hypothesis’ of social support [91]), including the chronic stress of physical disability itself. However, the number of studies which found insignificant or weak associations of social support with depression indicates limited support for this hypothesis. A review looking at social relationships and depression in the general population found 91.4% of studies to report a significant inverse association between social support and depression [92], in comparison we found only 59.0% of studies to report such an association. Moreover, the composite scores of social support which were often used in analysis integrated many distinct aspects of social support, often including items assessing received support alongside those assessing perceived support thus making it difficult to disentangle how different aspects of social support affected mental health and wellbeing. Inconsistent associations between social support and depression may therefore be explained by the potentially adverse effects of receiving social support in disability. Unwanted or unnecessary receipt of instrumental social support may have negative consequences among persons with disabilities, leading to reduced autonomy, self-worth and personal responsibility, all factors which are related to mental health and wellbeing [93]. Furthermore, when learning to interact with people in times of stress, such as during the adjustment and adaption to disability, high levels of social support could lead to higher levels of psychological distress. Moreover, increased support may be recognised or mobilised during times of distress, thus increasing the complexity of the relationships and the potential for reverse causality [94]. Evidence for the association of received instrumental support with mental health and wellbeing was lacking in this review, perhaps owing to the fact that long-term received support is often termed as ‘informal care’ in the literature.

The observation of inconclusive associations of social networks with indicators of mental health and wellbeing might be explained by the fact that social networks exert an indirect rather than direct effect on mental health and wellbeing [14]. This explanation is consistent with the conceptual model devised by Berkman et al. which suggests that social support is a resource attainable through access to the upstream factor of social networks [14]. This would suggest that social networks are important in their provision of social support but that their direct effect on mental health is minor. These results support the hypothesis by Cohen and Wills that qualitative support is more significant than social integration for persons under stress, i.e. persons with disabilities [91]. Additionally, extensive networks may not necessarily be supportive and members of social networks may be a source of stress or conflict [95].

### Potentials for future research

This review is based on an area of research that needs further development. One major limitation of this field of research concerns an inherent problem of the social relationships literature and refers to the potential tautology in the association between social relationships and distress. Although not significant in all cases, research indicates a clear trend towards an association between social relationships and mental health and/or wellbeing. As the majority of studies were cross-sectional, reverse causation in these findings cannot be excluded. It is evident that depressed or anxious people, for example, have trouble with social and interpersonal relationships and thus report lower social support or worse relationship quality [9]. To overcome this challenge and to meaningfully study this potentially tautological relationship, future studies should be based on longitudinal data and be grounded in well-reasoned theories that provide testable hypotheses. Ideally, a sound theoretical foundation should drive the instrumentation and the hypotheses, and ultimately, the interventions.

A further shortcoming of this area of research might be seen in the fact that several important aspects of social relationships such as loneliness, autonomy or reciprocity in social relationships remained unexplored and are therefore not tested in disability despite their importance in the general population [96, 97]. Moreover, potentially interesting concepts such as relationship quality, family functioning or negative social interactions were only tested in a marginally low number of studies. A greater insight into these domains would lead to better understanding of the complex roles different aspects of social relationships play in persons with disabilities. Moreover, it would be worthwhile to include different social relationship constructs within studies to investigate its potentially different effects on health.

A further and common limitation in disability research is the use of convenience samples recruited from specific inpatient or outpatient settings, or through patient organisations. This may limit the generalisability of the included studies by restricting the analysis to a niche group of individuals from a certain context [98]. Another methodological shortcoming concerns the quality of statistical analysis as we observed a restriction of analysis to bivariable associations without any adjustment for potential confounding in a number of studies. Also, we observe a lack of standardisation and homogeneity in the measurement of concepts, as for example 28 different instruments were used to measure social relationships, thus compromising the comparability of results.

### Limitations and strengths of this review

The study is subject to several limitations. Firstly, search terms for physical disability included only selected health conditions, meaning that the search was biased towards these conditions and may have missed papers addressing the theme of social relationships in other disabling conditions. Secondly, no meta-analysis could be performed due to the heterogeneity of measurements. Thirdly, as we only included studies using validated instruments, we may have missed new dimensions in research and focused too heavily on established areas in social relationship research, such as social support. Overall, 26 studies were excluded as they did not assess social relationships with a validated measurement instrument. We would therefore urge researchers to develop psychometrically tested instruments which give validated results for aspects of social relationships such as reciprocity. The search was also limited to papers published in peer-reviewed journals in English creating publication bias. Finally, the time frame of the literature search was restricted to 1995–2016. For feasibility reasons, we did not include previous research findings, but selective screening of the literature before 1995 confirmed consistency with the mainstream research represented in our review.

These limitations were balanced by several strengths. In our review, we structured a broad and heterogeneous field of research in terms of theoretical concepts of social relationships. Moreover, by distinguishing between mental health and wellbeing, we demonstrated the relevance of subjective appraisal of functioning and mood, aspects which are often unnoticed in traditional research on mental health. By summarising current evidence we were able to identify under and over researched areas in the field and at the same time demonstrated substantial methodological shortcomings. By doing so, we provide recommendations for promising future research developments. Despite predefined inclusion criteria (e.g. timeframe, language, and methodological issues), the literature search proved to be comprehensive, with the screening of over 5000 articles.

## Conclusion

We conclude that social relationships play an important role in mental health and wellbeing in persons with disabilities, although findings are less consistent than in the general population, strength of associations vary between constructs, and some important constructs such as loneliness, relationship quality or reciprocity are neglected in disability research. Integrating persons with disabilities into social networks is an important endeavour, however, it is of equal importance to strengthen the quality of their relationships and to tailor the level and kind of support to their needs. To promote mental health and wellbeing, rehabilitation professionals should support persons with disabilities and their significant others to ensure that high quality relationships are established and maintained, and that adequate support is available.

## Abbreviations

EURIDISS: European Research on Incapacitating Diseases and Social Support
ICD-10: International Classification of Diseases, version 10
PRISMA: Preferred Reporting Items for Systematic Reviews and Meta-Analyses
PTSD: Post-Traumatic Stress Disorder
SF-36: 36-Item Short Form Health Survey
STROBE: Strengthening the Reporting of Observational Studies in Epidemiology
