# Erratum to: Social relationships, mental health and wellbeing in physical disability: a systematic review

**DOI:** 10.1186/s12889-017-4448-8

**Published:** 2017-06-16

**Authors:** Hannah Tough, Johannes Siegrist, Christine Fekete

**Affiliations:** 1 Swiss Paraplegic Research, Guido A. Zäch Strasse 4, 6207 Nottwil, Lucerne, Switzerland; 2 Department of Health Sciences and Health Policy, University of Lucerne, Frohburgstrasse 3, P.O. Box 4466, 6002 Lucerne, Switzerland; 3 Senior Professorship ‘Work Stress Research’, Faculty of Medicine, University of Düsseldorf, Life-Science-Center, Merowingerplatz 1a, 40225 Düsseldorf, Germany

## Erratum

Following publication of this article [1], it has come to our attention that a part of Fig. 1 was included in error. The data “Other (17)” should not be included on the list of “Articles excluded based on abstract screening”. The original version of the article has been revised to reflect this.

**Fig. 1:**
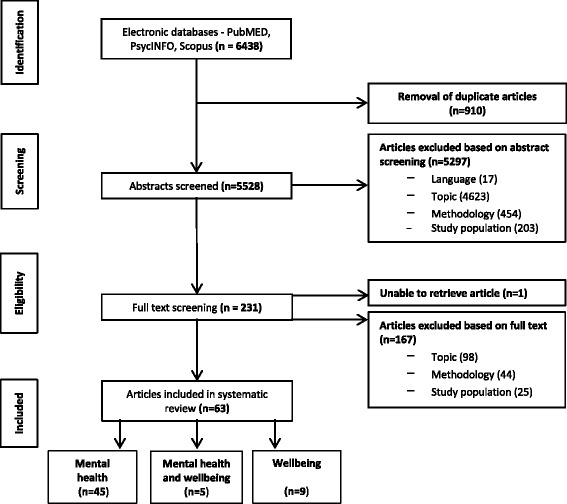
Flowchart of studies excluded and selected for systematic review
